# Starting school: educational development as a function of age of entry and prematurity

**DOI:** 10.1136/archdischild-2019-317124

**Published:** 2019-08-13

**Authors:** Katherine J Pettinger, Brian Kelly, Trevor A Sheldon, Mark Mon-Williams, John Wright, Liam J B Hill

**Affiliations:** 1 Bradford Institute for Health Research, Bradford, UK; 2 Department of Health Sciences, University of York, York, UK; 3 School of Psychology, University of Leeds, Leeds, UK

**Keywords:** preterm birth, prematurity, child development, born in Bradford, longitudinal

## Abstract

**Objective:**

To estimate the impact on early development of prematurity and summer birth and the potential ‘double disadvantage’ created by starting school a year earlier than anticipated during pregnancy, due to being born preterm.

**Design, setting and patients:**

We investigated the impact of gestational and school-entry age on the likelihood of failing to achieve a ‘Good Level of Development’ (GLD) on the Early Years Foundation Stage Profile in 5-year-old children born moderate-to-late preterm using data from the Born in Bradford longitudinal birth cohort. We used hierarchical logistic regression to control for chronological maturity, and perinatal and socioeconomic factors.

**Results:**

Gestational age and school-entry age were significant predictors of attaining a GLD in the 10 337 children who entered school in the correct academic year given their estimated date of delivery. The odds of not attaining a GLD increased by 1.09 (95% CI 1.06 to 1.11) for each successive week born early and by 1.17 for each month younger within the year group (95% CI 1.16 to 1.18). There was no interaction between these two effects. Children starting school a year earlier than anticipated during pregnancy were less likely to achieve a GLD compared with (1) other children born preterm (fully adjusted OR 5.51 (2.85–14.25)); (2) term summer births (3.02 (1.49–6.79)); and (3) preterm summer births who remained within their anticipated school-entry year (3.64 (1.27–11.48)).

**Conclusions:**

These results confirm the developmental risks faced by children born moderate-to-late preterm, and—for the first time—illustrate the increased risk associated with ‘double disadvantage’.

**What is already known on this topic?**

- Chronologically younger, summer-born children have worse educational outcomes compared with older children within their academic year.
- Prevalence of special educational needs is inversely proportional to gestational age.
- These differences have previously been demonstrated largely from 7 years old and upwards.

**What this study adds?**

- This is the first study to quantify the risk of poorer educational performance at 5 years of age if born preterm.
- Children born preterm who consequently enter school a year ‘early’ are ‘doubly disadvantaged’ due to reduced chronological and gestational age compared with their peers.
- School-entry age per se does not appear to moderate the risks posed by reduced gestational age.

## Objectives

Gestational age has been shown to be inversely proportional to the prevalence of special educational needs (SEN),^1^ and children born preterm (<37 weeks’ gestation) are at risk of developmental problems, as indexed by below-average attainment and higher levels of SEN at age 7 years and above.^2–4^ Intervention and support are often concentrated on children born extremely preterm though: in the UK enhanced surveillance and follow-up are only offered routinely for children born before 30 weeks’ gestation.^5^ This is despite evidence that children born moderate-to-late preterm (32–36 weeks’ gestation) are still at increased risk of poorer later development.^6^

Prematurity is one of many factors that determine the odds of a child showing good development. Another notable factor is a child’s age at the time they start school, determined in most countries by a cut-off linked to the start of the school year. Norbury *et al* 7 found that a child’s risk of not attaining a good level of development (GLD) at the end of the first year of mandatory schooling increased for every month born later in the academic year.^7^ For some children, the issue of school-entry age is connected to gestational age; premature birth can result in a child starting school a year earlier than expected during pregnancy. These children may face a ‘double disadvantage’ of relative immaturity at school entry due to both gestational and chronological age.

There is a need to better understand the impact that gestational age has on children’s developmental readiness for school in cases of prematurity and determine how this adds to, or interacts with, the effect of a child’s chronological maturity. Such insights would enable health and education providers to develop more evidence-based advice at this critical juncture and better support to children and their families.

We aimed to investigate whether there was an independent effect of both chronological age at school entry and gestational age and whether there was an interaction between them. We specifically sought to estimate the risks to early academic progress posed to children by entering school a year early due to preterm birth, by comparing them with other (1) children born preterm, (2) term summer births and (3) preterm summer births who remained within their anticipated academic year.

## Design and setting

We used the rich data set afforded by the ‘Born in Bradford’ (BiB) longitudinal birth cohort study to address these issues.^8^ We investigated the impact of gestational age on the likelihood of a child failing to achieve a ‘Good Level of Development’ following their first year of schooling (recorded using the Early Years Foundation Stage Profile, EYFSP).

## Participants

For inclusion in this analysis, BiB participants had to have linked data available from hospital maternity and local authority educational records, regarding their gestational age at birth and child development at 5 years old. A comparison of the linked data used from this subsample, relative to the greater cohort, is reported in table 1. Data from 10 390 children participating in BiB were used in our analyses.^8^ Participants had to have started school in the academic year expected given their birth date (ie, the September following their fourth birthday).

**Table 1:** Comparison of the demographics of all BiB participants with linked EYFSP and gestational age data compared with data available from the whole cohort

	EYFSP and gestational age n=10 390	Whole BiB cohort n=13 818
Educational factors		10 570*
EYFSP, n (%) ‘meeting GLD’†	6161 (59)	6253 (59)
English as an additional language, n (%)	4720 (45)	4788 (45)
Missing	111 (1)	114 (1)
Perinatal factors		13 525*
Gestational age (weeks), n (%)‡		
<32	81 (<1)	149 (1)
32–33	65 (<1)	110 (<1)
34–36	478 (5)	646 (5)
37–38	2355 (23)	3028 (22)
39–41	7269 (70)	9047 (67)
>42 (late term)	142 (1)	185 (1)
Month of birth, n (%) ‘Summer Born’§	3750 (36)	3816 (36)
Small for gestational age, n (%)	1449 (14)	1857 (14)
Large for gestational age, n (%)	711 (7)	954 (7)
Sex ratio (male:female)	49:51	52:48
Parity, median (range)	1 (0–10)	1 (0–10)
Socioeconomic factors		11 396*
Mother receiving means-tested benefits, n (%)	3683 (35)	4639 (41)
Missing	1859 (18)	39 (<1)
Maternal educational level (equivalised), n (%)		
Higher than A level	1991 (19)	2912 (26)
A level or equivalent	1277 (12)	1644 (14)
5 GCSEs or equivalent	2717 (26)	3488 (31)
<5 GCSEs or equivalent	1922 (18)	2453 (22)
Foreign unknown or other	523 (5)	741 (7)
Unable to answer	100 (<1)	128 (1)
Missing	1860 (18)	30 (<1)

*n within the whole cohort who consented and had linked records for this aspect of data collection.

†As defined by Cotzias and Whitehorn.^9^

‡Categories suggested by Jaekel *et al*.^11^

§Born between 1 April and 31 August.

BiB, Born in Bradford; EYFSP, Early Years Foundation Stage Profile; GCSE, General Certificate of Secondary Education; GLD, Good Level of Development.

### Exposures

Gestational age was measured in completed weeks. Gestational age was also compared with the estimated date of delivery to identify children for whom being born before term resulted in school entry an academic year earlier than anticipated during pregnancy, hereafter referred to as ‘Early Academic Starts’ (EAS).

## Main outcome measures

The GLD measure from the EYFSP is the most widely used single measure of child development at 5 years old in the UK.^9^ An EYFSP assessment is completed for every child in state-funded, early-years education in England during the final term of the academic year in which they turn 5. Teachers assess children’s progress in each of the learning areas as ‘emerging’, ‘expected’ or ‘exceeding’. In order to be classified as reaching a ‘Good Level of Development’, they must achieve either ‘expected’ or exceeding’ in the prime learning areas of ‘personal, social and emotional development’, ‘physical development’, ‘communication and language’, as well as mathematics and literacy.^9^ Previous work has shown that summer-born children tend to score worse on these EYFSP items.7 9=

### Explanatory variables

#### Perinatal factors

The mother’s parity and child’s month of birth, sex (male or female), and whether at birth they were classified as ‘small’ or ‘large’ for gestational age (SGA or LGA) were obtained from Bradford Royal Infirmary maternity records. ‘Small’ and ‘large’ categories were defined as falling either below the 10th percentile or above the 90th, respectively, on the WHO (UK) fetal growth charts for sex and gestational week at birth. This measure was only available for singleton births. Month of birth was transformed to reflect the month of birth relative to the start of the academic year (eg, September=1st month…August=12th month) and is hereafter referred to as ‘*Academic* Month of Birth’ (AMoB).

#### Socioeconomic and educational factors

Socioeconomic influences were measured using the mother’s self-reported highest level of educational qualification (maternal highest qualification, MHQ), equivalised if educated abroad, and whether they received means-tested benefits (MTB). Both were recorded as part of the BiB cohort maternal baseline questionnaire.^8^ These criteria have been used to estimate socioeconomic status within the BiB cohort in previously published research.^10^ From the local authority educational records, a measure of whether a child was known to speak English as an additional language (EAL) was also captured (coded as: yes/no/don’t know).

### Statistical analysis

Logistic regression, using a generalised linear modelling (GLM) method (in R V.3.4.3), was used to estimate the relationship between gestational age (in completed weeks) and a child’s likelihood of not achieving a GLD on the EYFSP. An initial set of exploratory analyses (see online supplementary table S1) fitted this relationship with linear (online supplementary table S1, step 1) and then polynomial, quadratic and then cubic terms (steps 2 and 3, respectively) because previous research has suggested gestation has a non-linear relationship with certain developmental outcomes.^11^

10.1136/archdischild-2019-317124.supp1 Supplementary data

A first set of analyses looked specifically at predictors of not achieving a GLD within children whose date of birth had not resulted in them starting school a year earlier than they otherwise would have had they been born at term. In this sample, regression models tested the effect of gestational age after controlling for a child’s AMoB (table 3, step 2), then in a subsequent third step tested whether there was a significant moderating effect of AMoB on gestational age. A further two adjusted versions of this analysis were run (see online supplementary table S2, steps 2 and 3). In the first of these, additional perinatal covariates (parity, gender, SGA and LGA) were controlled for alongside AMoB to see if the effects of gestational age remained after controlling for these factors. In the second, the model adjusted for these perinatal factors (parity, gender, SGA and LGA) as well as additional socioeconomic and educational factors (MHQ, MTB and EAL).

**Table 3:** Hierarchical logistic regression of effects of gestational age (in weeks) on failing to attain a Good Level of Development on the EYFSP after controlling and moderating for the additional effects of academic month of birth

Variable	Step 1	Step 2	Step 3
	OR (95% CI)	OR (95% CI)	OR (95% CI)
Academic month of birth	1.17 (1.15 to 1.18)***	1.17 (1.16 to 1.18)***	1.17 (1.16 to 1.18)***
Gestational age (in weeks)		1.09 (1.06 to 1.11)***	1.08 (1.06 to 1.11)***
Interaction†			0.99 (0.99 to 1.01)
R^2^ (Hosmer-Lemeshow)	0.047	0.050	0.050
R^2^ (Cox-Snell)	0.061	0.065	0.065
R^2^ (Nagelkerke)	0.082	0.088	0.088
χ^2^‡	651.7***	47.2***	0.2

n=10 337.

*P<.05, **p<0.01, ***p<0.001.

**†**Gestational age × academic month of birth.

‡Significance denotes *change* in model fit from previous step in the hierarchical model.

EYFSP, Early Years Foundation Stage Profile.

A second set of analyses tested for specific differences in the likelihood of not achieving a GLD in the EAS cases, compared with (1) preterm children *not* born during the summer months, June–August, hereafter referred to as ‘summer-born’ (ie, controlling for gestational but not chronological maturity); (2) term summer-born children (ie, controlling for chronological but not gestational maturity); and (3) preterm summer-born children whose year of school entry was unaffected by prematurity (ie, controlling for both types of maturity).

As with the preceding analyses, relationships between early school entry and the GLD outcome were first investigated as a univariable analysis (step 1 in online supplementary tables S3–S5) before successively adjusting for further perinatal (steps 2) and socioeconomic and educational covariates (steps 3). The only differences were that in the models comparing EAS against other preterm children, gestational age was entered as an additional covariate alongside the other perinatal factors, while in the model comparing EAS against other (non-premature) summer-born children AMoB was entered. Prior to conducting adjusted analyses, to mitigate for loss of power in these analyses created by missing at random data, values for missing data within covariates were estimated using a multiple imputation chained equation technique, implemented using the  Multivariate Imputation by Chained Equations (MICE) package in R.^12^

## Results

Seven children failed to meet the inclusion criteria of starting school in the standard academic year anticipated by their birth date. None of these children were preterm. All seven children started school a year late, which is usually only permitted by local authorities in exceptional circumstances, as suggested by these excluded cases all being recorded as having SEN.

All the children who entered school an academic year earlier than anticipated during pregnancy (EAS) were categorically ‘preterm births’, representing 8.5% of the preterm births in the sample (53 out of 624). No children were found to start ‘late’ due to being born after their estimated date of delivery. Five cases of EAS were lost to further analysis because of complete but uncategorisable responses for covariates (eg, a response of ‘foreign unknown’ for MHQ). Comparison of the demographic make-up of the 48 remaining cases of EAS with the other groups is presented in table 2.

**Table 2:** Comparison of demographics of children who enter school a year early due to premature birth ‘Early Academic Starts’ versus preterm births outwith June–August, term June–August births and preterm births in June–August not entering school early

	Early Academic Starts n=48	Preterm not June–August births n=457	June–August term births n=1972	Preterm + June–August not EAS n=64
Educational factors				
EYFSP, n (%) ‘meeting GLD’*	9 (19)	239 (52)	841 (43)	24 (38)
English as an additional language, n (%)	19 (40)	191 (42)	973 (49)	40 (63)
Perinatal factors				
Gestational age (weeks), n (%)†				
<32	10 (21)	61 (13)	–	6 (9)
32–33	6 (13)	49 (11)	–	4 (6)
34–36	32 (67)	347 (78)	–	54 (84)
37–38	–	–	458 (23)	–
39–41	–	–	1478 (75)	–
>42 (late term)	–	–	36 (2)	–
Small for gestational age, n (%)‡	5 (10)	54 (12)	255 (13)	7 (11)
Large for gestational age, n (%)‡	2 (4)	42 (9)	144 (7)	4 (6)
Sex ratio (male:female)	50:50	47:53	47:53	47:53
Parity, median (range)‡	1 (0–3)	1 (0–8)	1 (0–8)	1 (0–5)
Socioeconomic factors				
Mother receiving means-tested benefits, n (%)‡	24 (50)	197 (43)	852 (43)	27 (42)
Maternal educational level (equivalised), n (%)‡				
Higher than A level	11 (23)	119 (26)	520 (26)	14 (22)
A level or equivalent	9 (19)	77 (17)	302 (15)	11 (17)
5 GCSEs or equivalent	17 (35)	131 (29)	690 (35)	23 (38)
<5 GCSEs or equivalent	11 (23)	130 (28)	460 (23)	16 (25)

*As defined by Cotzias and Whitehorn.^9^

†Categories suggested by Jaekel *et al*.^11^

‡Contains imputed values for missing data.

EAS, Early Academic Starts; EYFSP, Early Years Foundation Stage Profile; GCSE, General Certificate of Secondary Education; GLD, Good Level of Development.

### Gestational age and AMoB

The most parsimonious model fit for gestational age as predictor, in this data set, was found to be linear. There were no statistically significant improvements in model fit from estimating further polynomial parameters (tested using −2 log likelihood ratio tests, all p>0.05). Thus all subsequent analyses modelled gestational age as a linear predictor of GLD. Full tabular summaries of this and further analyses are presented across table 3 and online supplementary tables S1–S5.

Logistic regression (table 3) showed that gestational age, in weeks, was a significant predictor of whether a child attained a GLD on the EYFSP (χ^2^(1)=651.7, p<0.001). This effect persisted after controlling for the significant effect of AMoB (χ^2^(1)=47.2, p<0.001). ORs indicated that for each successive week that a child was born early, their odds of *not* attaining a GLD increased by 1.09 (95% CI 1.06 to 1.11). In addition, for every month later in the academic year the child was born, these odds also increased by 1.17 (95% CI 1.16 to 1.18). However, no significant improvement in model fit arose from adding an interaction between gestational age and AMoB to this model (χ^2^(1)=0.2, p=0.7), suggesting AMoB did *not* moderate the risks posed by reduced gestational age.

**Figure 1:**
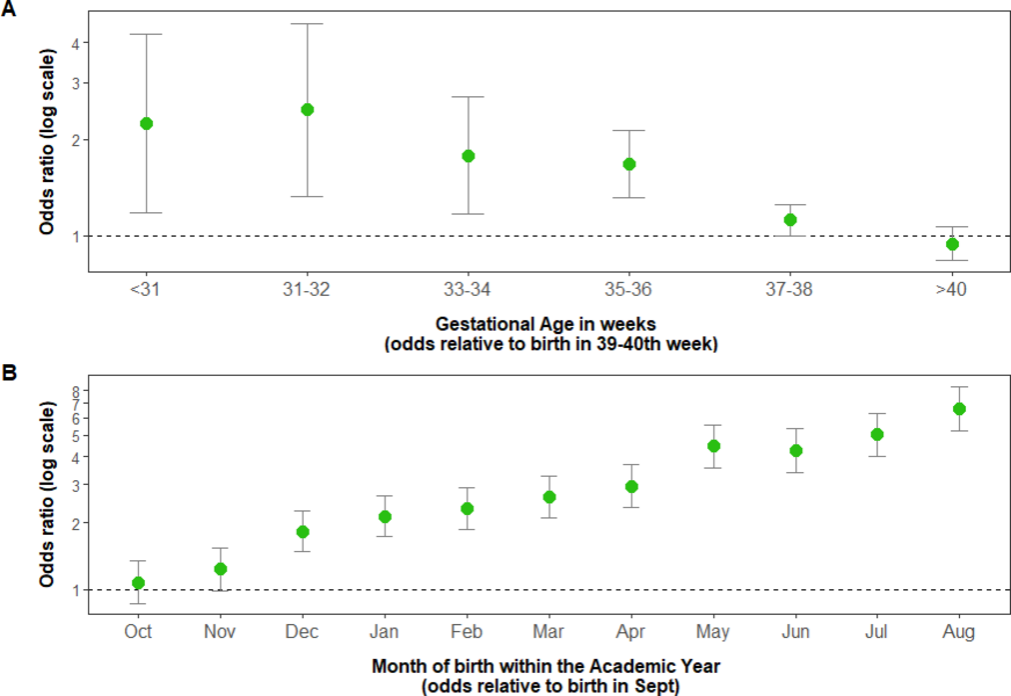
The effects of (A) gestational age in weeks and (B) academic month of birth on the odds of failing to achieve a Good Level of Development after fully adjusting for covariates.

Adjustment for additional covariates did not materially affect the estimates of these effects, with the odds for both the effects of gestational age 1.09 (95% CI 1.06 to 1.12) and AMoB 1.19 (95% CI 1.17 to 1.20) still comparable in magnitude. Illustrations of these effects, after fully adjusting for covariates, are presented in figure 1.

### EAS versus comparator groups

**Figure 2:**
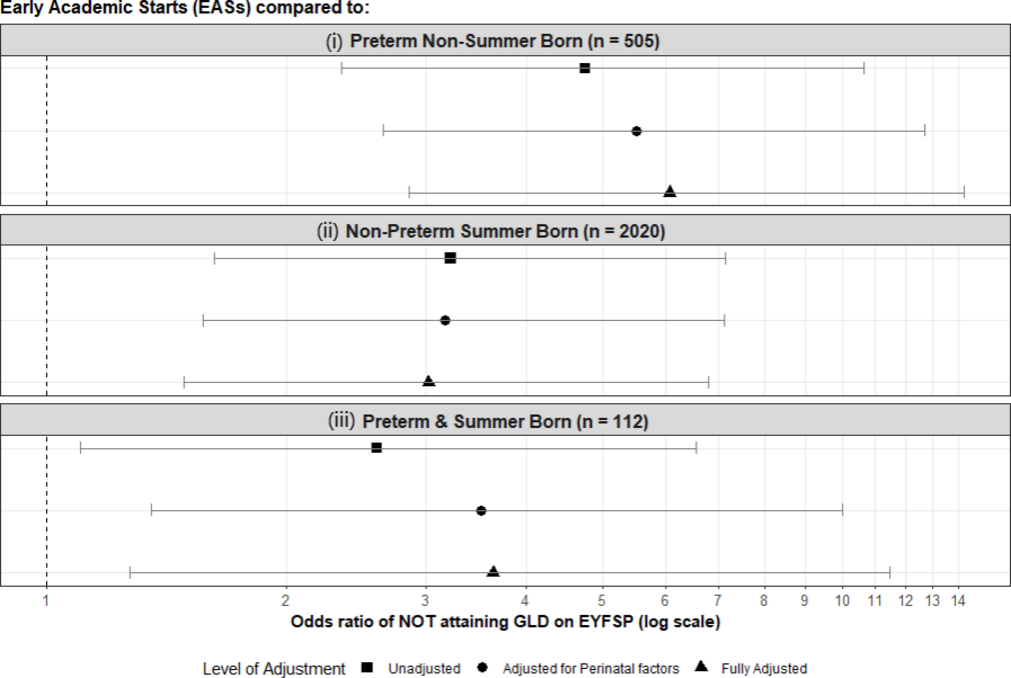
The odds of failing to attain a GLD due to being an EAS compared with (i) preterm non-summer-born children, (ii) non-preterm summer-born children and (iii) preterm summer-born children who remained within the academic year anticipated during pregnancy. EAS, Early Academic Starts; EYFSP, Early Years Foundation Stage Profile; GLD, Good Level of Development.

Children starting school a school year earlier than anticipated during pregnancy were less likely to achieve a GLD compared with (1) other children born preterm (fully adjusted OR 5.51 (2.85–14.25)); (2) term summer births (fully adjusted OR 3.02 (1.49–6.79)); and (3) preterm summer births who remained within their anticipated school year (fully adjusted OR 3.64 (1.27–11.48)). These effects again remained after adjustment (see figure 2 and online supplementary tables S3–S5).

## Discussion

Our findings indicate differences in the odds that 5-year-old children will exhibit the level of development expected by the national education system due to both gestational and school-entry age. The results are consistent with earlier research^7^ in that the odds of not achieving a GLD almost double in children born in the final (vs the first) month of an academic school year. The incremental effect of gestational age each week was such that for a child born at 34 weeks’ gestation, there was an approximate doubling of the odds of them not attaining the level of expected development, compared with a child born at term.

In particular, children who entered school a year early due to being born premature appeared to be at substantial disadvantage. This group’s odds of not showing a GLD were more than three-and-a-half times greater than even the summer-born preterm children who did *not* start school a year early.

The specific ‘double disadvantage’ of prematurity causing a child to be chronologically young within the school year has rarely been considered in previous research. Odd *et al* 4 report a relationship between preterm birth and poorer academic attainment at 7 and 16 years of age, which disappeared when children who started school a year early due to preterm birth were excluded from analysis. Johnson *et al* 2 showed that children born ≤25 weeks’ gestation who entered school a year earlier than anticipated were more likely to have SEN but did not show differences on standardised academic test scores at age 11 years.^2^ Jaekel *et al*’s^13^ studied a cohort of German schoolchildren, finding no differences between children with and without delayed school entry on attainment at the end of the first year of school. However, those children whose school entry was deferred performed worse on standardised assessments at age 8 years. This may reflect a negative impact of delayed entry or that the most profoundly affected children are over-represented in the delayed entry group.

Further research is needed to gain a clearer understanding of why a symbiotic relationship between preterm birth and school-entry age exists in EAS. Norbury *et al* 7 report that, in conjunction with being less likely to reach the expected level on the EYFSP, the youngest children in a school year typically also exhibit more inter-related behavioural and language difficulties. Such difficulties also co-occur more frequently in children born moderate-to-late preterm in early childhood.^14^ Thus, it may be that the increased liklihood of difficulties faced by children who start school early due to premature birth relate to these risk factors converging.

Meanwhile, a recent study using parental questionnaires showed only a small proportion of moderate-to-late preterm children had problems typically associated with prematurity.^15^ Our findings suggest the contrary and add weight to arguments for more support for moderate-to-late preterm children, previously presumed to require no additional surveillance.^5^ Indeed, these results illustrate differences in educationally relevant aspects of a child’s development at an earlier age than previous research.^2 4^ As part of the BiB cohort study, we will be following these children’s developmental progress over their life course and investigating the longer term effects on educational and economic attainment.

### Implications for practice and policy

The assessment of childhood development we used can be treated as an estimate of a child’s capacity to cope with the transition into formal education^9^; thus, our findings suggest the need for a more proactive approach by health and education professionals in supporting gestationally immature children through this transition. Possible methods for doing this include providing learning resources to teachers to help them support children born preterm in the classroom (such as those developed by Johnson and colleagues^16^), tailored advice to families, and greater use of routine data linkage to more easily allow at-risk children to be monitored and supported longitudinally, across health and education services. Such interventions may be vitally important in mitigating the cascading risks that preterm birth poses later for mental health, cognitive development and academic attainment.^17^

We found no evidence of age *within* a given academic year interacting with the preterm risk, and while this was not an analysis of the direct effect of delaying entry (this analysis was not possible due to too few cases of delayed entry in the cohort), this lack of a moderating effect may suggest that simply delaying the age of school entry per se may not be the best way to support children born prematurely.

## Conclusions

We found strong independent effects of chronological age and prematurity on a child’s developmental readiness for schooling and that children starting school a year earlier than anticipated during pregnancy *are* ‘doubly disadvantaged’. This work provides further evidence of the interplay between health and education and is intended as a first step away from arbitrary decision making, instead allowing health and education departments to work together to guide where resources should be directed. We hope this will become an example of progress towards evidence-based policy making—an approach already being adopted by Bradford local authority.
