# Supplementary material for: Starting school: educational development as a function of age of entry and prematurity
Source: Arch Dis Child. 2019 Aug 13;105(2):160–5. doi: 10.1136/archdischild-2019-317124 (PMC7025727; doi:10.1136/archdischild-2019-317124)
Supplement: Supplementary data [file archdischild-2019-317124supp001.pdf]

Table S1. Hierarchical Logistic Regression of effects of Gestational Age (in weeks) on failing to attain a Good Level of Development on the EYFSP fitted as a linear, quadratic or cubic function

	Step 1	Step 2	Step 3
**Variable**	OR (95% CI)	OR (95% CI)	OR (95% CI)
**Gestational Age** (in Weeks)			
**Linear**	1.06 (1.04-1.09)***	1.04 (1.01-1.08)*	1.03 (0.99-1.08)
**Quadratic**		1.00 (1.00-1.01)	1.01 (0.99-1.02)
**Cubic**			1.00 (1.00-1.01)
R^2 (Hosmer-Lemeshow)	.002	.002	.002
R^2 (Cox-Snell)	.003	.003	.003
R^2 (Nagelkerke)	.004	.004	.004
$\chi^{2\,\dagger}$	27.7***	1.9	0.4

Notes: N = 10337

† Significance denotes *change* in model fit from previous step in the hierarchical model

‡ Gestation Age x Academic Month of Birth

*p<.05, **p<.01, ***p<.001

Table S2. Hierarchical Logistic Regression of effects of Gestational Age on failing to attain a Good Level of Development on the EYFSP, with adjustment for Perinatal, and Education and Socioeconomic covariates.

	Step 1	Step 2	Step 3
**Variable**	OR (95% CI)	OR (95% CI)	OR (95% CI)
**Gestational Age** (in Weeks)	1.06 (1.04-1.09)***	1.06 (1.04-1.09)***	1.09 (1.06-1.12)***
Perinatal			
**Registered Parity**		1.12 (1.09-1.16)***	1.00 (0.97-1.04)
**Sex** [Male]		2.08 (1.91-2.27)***	2.18 (2.00-2.39)***
**Large for Gestational Age** [Yes]		0.80 (0.67-0.95)*	0.88 (0.72-1.04)
**Small for Gestational Age** [Yes]		1.64 (1.45-1.85)***	1.57 (1.38-1.77)***
**Academic Month of Birth**		1.17 (1.16-1.19)***	1.19 (1.17-1.20)***
Education & Socioeconomic			
**English as Additional Language** [Yes]			1.28 (1.17-1.41)***
**Means Tested Benefit** [Yes]			1.24 (1.13-1.37)***
**Maternal Highest Qualification**			
GCSE			0.63 (0.56-0.70)***
A-level			0.47 (0.42-0.56)***
Higher than A-Level			0.32 (0.28-0.37)***
R^2 (Hosmer-Lemeshow)	.002	.080	.111
R^2 (Cox-Snell)	.003	.102	.139
R^2 (Nagelkerke)	.004	.138	.188
$\chi^{2\,\dagger}$	26.9***	46.8***	44.0***

Notes: N = 9552, values in square-brackets indicate category tested
† Significance denotes *change* in model fit from previous step in the hierarchical model
*p<.05, **p<.01, ***p<.001

Table S3. Hierarchical Logistic Regression Model of predictors of failing to attain a Good Level of Development on the EYFSP due to being an Early Academic Start, compared to other Pre-term births that were not Summer Born

Variable	Step 1<br>OR (95% CI)	Step 2<br>OR (95% CI)	Step 3<br>OR (95% CI)
**EAS vs. Pre-term Non Summer Born**	4.75 (2.35-10.66)***	5.51 (2.65-12.69)***	6.07 (2.85-14.25)***
Perinatal			
**Registered Parity**		1.29 (1.11-1.50)***	1.17 (0.99-1.39)
**Sex** [Male]		2.42 (1.65-3.56)***	2.86 (1.91-4.34)***
**Large for Gestational Age** [Yes]		0.67 (0.35-1.31)	0.61 (0.30-1.23)
**Small for Gestational Age** [Yes]		4.40 (2.33-8.80)***	5.87 (2.94-12.44)***
**Gestational Age** (in Weeks)		1.09 (1.01-1.19)*	1.08 (0.99-1.18)
Education & Socioeconomic			
**English as Additional Language** [Yes]			1.18 (0.79-1.77)
**Means Tested Benefit** [Yes]			1.16 (0.74-1.81)
**Maternal Highest Qualification**			
GCSE			0.52 (0.31-0.89)*
A-level			0.42 (0.23-0.77)**
Higher than A-Level			0.16 (0.09-0.29)***
R^2 (Hosmer-Lemeshow)	.030	.106	.171
R^2 (Cox-Snell)	.041	.137	.211
R^2 (Nagelkerke)	.054	.183	.281
$\chi^{2\,\dagger}$	21.0***	23.2***	24.4***

Notes: N = 505. EAS = Early Academic Start, values in square-brackets indicate category tested
† Significance denotes *change* in model fit from previous step in the hierarchical model
*p<.05, **p<.01, ***p<.001

Table S4. Hierarchical Logistic Regression Model of predictors of failing to attain a Good Level of Development on the EYFSP due to being an Early Academic Start, compared to other Summer Born non Pre-term births

	Step 1	Step 2	Step 3
**Variable**	OR (95% CI)	OR (95% CI)	OR (95% CI)
**EAS vs. Summer Born non Pre-term**	3.22 (1.63-7.13)**	3.17 (1.57-7.11)**	3.02 (1.49-6.79)**
Perinatal			
**Registered Parity**		1.10 (1.02-1.18)*	0.97 (0.89-1.04)
**Sex** [Male]		2.27 (1.89-2.73)***	2.35 (1.95-2.84)***
**Large for Gestational Age** [Yes]		1.08 (0.76-1.54)	1.15 (0.80-1.67)
**Small for Gestational Age** [Yes]		1.62 (1.22-2.16)***	1.58 (1.19-2.12)**
**Academic Month of Birth**		1.20 (1.07-1.34)**	1.24 (1.11-1.40)***
Education & Socioeconomic			
**English as Additional Language** [Yes]			1.34 (1.10-1.63)**
**Means Tested Benefit** [Yes]			1.22 (0.99-1.51)
**Maternal Highest Qualification**			
GCSE			0.57 (0.43-0.74)***
A-level			0.36 (0.26-0.49)***
Higher than A-Level			0.30 (0.23-0.40)***
R^2 (Hosmer-Lemeshow)	.004	.043	.081
R^2 (Cox-Snell)	.006	.057	.105
R^2 (Nagelkerke)	.008	.077	.141
$\chi^{2\dagger}$	12.1***	11.1***	9.9**

Notes: N = 2020 . EAS = Early Academic Start, values in square-brackets indicate category tested
† Significance denotes *change* in model fit from previous step in the hierarchical model
*p<.05, **p<.01, ***p<.001

Table S5. Hierarchical Logistic Regression Model of predictors of failing to attain a Good Level of Development on the EYFSP due to being an Early Academic Start, compared to other Summer Born Pre-term births

	Step 1	Step 2	Step 3
**Variable**	OR (95% CI)	OR (95% CI)	OR (95% CI)
**EAS vs. Summer Born & Pre-term**	2.60 (1.10-6.56)*	3.51 (1.35-10.02)*	3.64 (1.27-11.48)*
Perinatal			
**Registered Parity**		1.11 (0.76-1.66)	1.23 (0.71-1.83)
**Sex** [Male]		2.73 (1.15-6.76)*	3.10 (1.22-8.37)*
**Large for Gestational Age** [Yes]		1.04 (0.17-8.47)	1.12 (0.17-10.07)
**Small for Gestational Age** [Yes]		2.38 (0.54-16.94)	2.15 (0.40-17.06)
**Gestational Age** (in Weeks)		0.90 (0.75-1.66)	0.90 (0.74-1.10)
Education & Socioeconomic			
**English as Additional Language** [Yes]			0.85 (0.32-2.25)
**Means Tested Benefit** [Yes]			0.93 (0.27-3.12)
**Maternal Highest Qualification**			
GCSE			1.19 (0.29-4.68)
A-level			0.31 (0.06-1.50)
Higher than A-Level			0.35 (0.07-1.68)
R^2 (Hosmer-Lemeshow)	.035	.094	.147
R^2 (Cox-Snell)	.042	.108	.163
R^2 (Nagelkerke)	.060	.153	.232
$\chi^{2\dagger}$	4.8*	6.8**	5.9*

Notes: N = 112. EAS = Early Academic Start, values in square-brackets indicate category tested
[†] Significance denotes *change* in model fit from previous step in the hierarchical model
*p<.05, **p<.01, ***p<.001
